# Cultivation of Bdelloid Rotifer *Adineta vaga* with Synthetic Medium and Characterization of Associated Bacteria

**DOI:** 10.3390/biology14111507

**Published:** 2025-10-28

**Authors:** Wenbo Wang, Zhili He, Qing Wang, Yufeng Yang

**Affiliations:** 1 Institute of Hydrobiology, Jinan University, The Southern Marine Science and Engineering Guangdong Laboratory (Zhuhai), Guangzhou 510632, China; wwb129@163.com (W.W.); wq2010@jnu.edu.cn (Q.W.); 2 The Southern Marine Science and Engineering Guangdong Laboratory (Zhuhai), Zhuhai 519080, China; hezhili@sml-zhuhai.cn

**Keywords:** Bdelloidea, synthetic rotifer medium, associated microorganism, method, antibiotic

## Abstract

**Simple Summary:**

Bdelloid rotifers are important model organisms for evolutionary and ecological research, yet their laboratory cultivation has traditionally relied on nutritionally variable natural food sources, limiting mechanistic studies of host–microbe interactions. This study developed a chemically defined Synthetic Rotifer Medium (SRM) that supports population growth of *Adineta vaga* comparable to traditional food-based systems. Using this standardized platform, we isolated and identified 20 bacterial strains from *A. vaga*, comprising 11 endozoic and 9 epizoic isolates. Antibiotic treatment experiments demonstrated that bacterial clearance remained incomplete while simultaneously reducing rotifer population growth. This work establishes key resources—a defined cultivation medium and a bacterial strain collection—which provide a foundation for future investigations into rotifer-microbial interactions and coevolutionary processes.

**Abstract:**

Bdelloid rotifers are model organisms for evolutionary genetics; however, their laboratory cultivation has been limited to traditional systems that require natural food sources (e.g., lettuce juice, bacteria, or yeast) of undefined composition. This constraint impedes mechanistic studies of rotifer–microbe interactions and genetic evolution. We developed a synthetic rotifer medium (SRM) that enables axenic cultivation of *Adineta vaga*, the most commonly used model species of bdelloid rotifers in the laboratory, as a chemically controlled alternative. *A. vaga* reached a population density of 357 ± 19.95 ind./mL with a specific growth rate of 0.2131 ± 0.003 over 20 days in SRM, achieving parity with traditional food-supplemented systems while eliminating compositional variability. We further isolated 20 bacterial strains associated with SRM-cultured *A. vaga*, which were affiliated with two genera (*Pseudomonas* and *Aquincola*) on the body surface, as well as four genera (*Lentzea*, *Streptomyces*, *Sphingomonas* and *Spirosoma*) and one family (Burkholderiaceae) inside *A. vaga*. Additionally, the addition of low-concentration antibiotics over 20 days reduced the population size or specific growth rate of *A. vaga*, and cannot fully eliminate the associated bacteria. This study established the first nutritionally autonomous, compositionally stable culture system for bdelloids, enabling precise investigation of rotifer–microbe coevolution and functional genetics.

## 1. Introduction

Bdelloid rotifers (Rotifera, Bdelloidea) are one of the main microscopic invertebrates in aquatic/limno-terrestrial habitats, most of which are between 150–700 μm in length [1]. Several distinct biological traits, including parthenogenesis, anhydrobiosis, and horizontal gene transfer, characterize them. As one of the oldest and most diverse clades of asexually evolving metazoans, bdelloid rotifers represent an evolutionary enigma, often referred to as the “sleeping beauty” of the animal kingdom, that has persisted via obligate asexuality for over 80 million years. Their long-term evolutionary success challenges conventional theories of the adaptive supremacy of sexual reproduction [2]. Their strong environmental adaptability and cryptobiotic capacity, including survival under desiccation, cryobiosis, and ionizing radiation (>500 Gy), make them model organisms for developmental biology in space [3,4,5]. Horizontal gene transfer (HGT) from non-metazoan organisms (bacteria, fungi and plants) confers novel stress-responsive traits such as antifungal defenses [6] and cryoprotective adaptations [7]. Given their unique position in evolutionary biology, bdelloids are emerging as tractable systems for studying genetic differentiation, adaptive evolution, biological development, aging, and various ecological experiments. Furthermore, bdelloid rotifers are highly abundant in natural environments, with densities reaching up to 2.1 × 10^6^ ind./m^2^ in soil habitat [8], making them the third most abundant group behind Nematozoa and Arthropoda [9]. They serve as key intermediaries in microbial food webs [9]. Because of their ecological relevance and documented sensitivity to environmental change, shifts in rotifer populations and physiology can indicate alterations in water and soil quality and broader ecosystem health [10]. Therefore, they are well-suited for applications in environmental monitoring and ecotoxicological research [11].

The above research typically requires large populations (~1000–50,000 ind.) for high-quality omics [12,13], necessitating efficient laboratory cultivation. Current methods rely on traditional systems supplemented with nutritionally variable natural foods (e.g., lettuce juice, bacteria, yeast, microalgae, flour, and other foods) [14,15], which introduce several critical limitations. (1) The complex and undefined composition of natural foods introduces uncontrolled metabolic byproducts, obscuring the true nutrient requirements of rotifers. (2) The nutritional quality of natural food sources, such as lettuce juice, is highly variable and can degrade over time during cultivation. Additionally, maintaining consistency across different batches of lettuce juice is difficult, leading to potential variability in culture outcomes. (3) Natural foods, such as microalgae, often carry associated microorganisms, introducing microbial contamination into rotifer cultures, which can alter rotifer physiology. (4) The cultivation of these biological foods is time-consuming, resulting in operational inefficiency. These methodological limitations become particularly serious in mechanistic research on bdelloid rotifer–microbe interactions and their environmental adaptability, underscoring the importance of precise quantification of substrate utilization, interspecies metabolite exchange, and nutrient cycling pathways. Therefore, it is necessary to establish a chemically defined synthetic rotifer culture medium that does not contain natural foods.

Bdelloid rotifers are closely related to microorganisms that may serve as nutritional sources. As basal consumers in aquatic and terrestrial ecosystems, bdelloid rotifers primarily feed on bacteria. For example, a natural population of *Habrotrocha thienemanni* (56,800 ind./L) clears bacteria at rates ranging from 981 to 5170 mL/L/day [16]. Dekić et al. [17] found that bdelloid rotifer *Adineta vaga* could exert a strong grazing pressure on bacteria and bacterial communities, especially in oligotrophic environments. The strong grazing pressure exerted by bdelloid rotifers on microorganisms may, in turn, influence biogeochemical cycles in natural ecosystems [16]. In addition, bdelloid rotifers can inhibit bacterial production of extracellular polymeric substances [15].

At the genetic level, bacteria-derived genes have been detected in bdelloid rotifer genomes [6,18]. Wilson et al. [18] suggested that bacteria could contribute 53% of the horizontal gene transfer candidate (HGTc) genes in bdelloid rotifers. These bacteria-derived genes can function alongside bdelloid genes, improving the survival rates of bdelloid rotifers, such as the production of specific peptides that resist fungal pathogens [6,7].

Despite this interdependence, rotifer-associated bacterial communities remain poorly characterized. Recent studies have preliminarily characterized bdelloid rotifer-associated bacterial communities using 16S rRNA amplicon sequencing [19,20]. However, the ecological roles of these bacteria and the mechanistic pathways through which they influence rotifer survival and environmental adaptation remain unclear. Critically, the absence of associated bacterial strains has hindered mechanistic investigations of rotifer–microbe interactions. Such constraints necessitate direct experimental validation through controlled co-cultures of axenic bdelloid rotifer hosts with defined bacterial strains in a synthetic culture medium. Therefore, we need to collect and establish rotifer-associated bacterial strain resources, develop gnotobiotic bdelloid models, and conduct interaction assays in chemically defined synthetic media. These procedures represent an essential pathway for elucidating the symbiotic mechanisms between rotifers and microorganisms.

*A. vaga* was selected as the experimental subject in this study due to its well-established status as a model organism for investigating HGT and asexual evolution [13,21,22]. It is one of the few bdelloid rotifers with a fully sequenced genome, providing a solid genetic foundation for mechanistic research [13]. Furthermore, *A. vaga* is distributed globally and is easy to collect. Established cultivation research using natural foods also enables a direct comparison with our new medium [14]. The aims of the present study were to (1) develop a chemically defined synthetic rotifer medium (SRM) without natural food for *A. vaga*, providing a key tool for axenic cultivation and mechanistic research; (2) isolate and obtain pure cultures of bacteria associated with *A. vaga*, thereby establishing a microbial resource for studying rotifer–bacteria interactions; (3) evaluate the responses of *A. vaga* populations and their associated bacteria to antibiotic treatments, aiming of obtaining axenic bdelloid rotifers. By fulfilling these aims, this study pioneers new avenues for research on rotifer–microbe coevolution, metabolic exchange, and the genetic basis of environmental adaptation. The novel medium and associated findings can potentially be extended to diverse zooplankton taxa, including *Cladocera*, *Copepoda*, and *Monogononta rotifers*.

## 2. Materials and Methods

### 2.1. Bdelloid Rotifer

The bdelloid rotifer *A. vaga* was isolated from moss growing on the soil surface in the Qiao Island Mangrove Forest Nature Reserve (Guangdong, China), following the method described by Wang et al. [23]. Eighteen *A. vaga* individuals were selected, rinsed five times with sterile water separately, and transferred to a 24-well plate containing 1 mL sterile water for interim cultivation.

### 2.2. Culture Media and Culture Methods

The initial culture was established from a single *A. vaga* individual and tested in carbohydrate-amended basal media, including Blue-Green 11 (BG-11) medium, Environmental Protection Agency (EPA) algal medium, and nematode growth medium (NGM), with six replicates for each group. Preliminary screening revealed that BG-11-derived modifications supported viable offspring production in target rotifers. Consequently, the SRM was designed with fully defined chemical constituents based on the BG11 framework. For comparative assessment, traditional food-supplemented systems (lettuce juice and wheat flour media) were used as controls, given their undefined compositional profiles.

(1) SRM:

SRM was developed as a new medium based on BG-11 medium. Metazoan invertebrates such as rotifers have substantially higher nutritional and metabolic demands than phytoplankton. Several organic components have been introduced to satisfy these requirements. A combination of carbohydrates (glucose, sucrose, and fructose) was used as an energy sources. Glucose serves as a readily utilizable carbon source for immediate metabolic needs, and sucrose acts as a disaccharide that provides sustained carbon release, whereas fructose offers an alternative metabolic pathway. This multi-carbohydrate strategy enhances the robustness of the medium by ensuring a diverse and continuous energy supply, thereby reducing the risk of metabolic bottlenecks associated with single carbon sources. Vitamins B1 (thiamine), B6 (pyridoxine), and B7 (biotin) are well-established essential micronutrients in aquatic animal culture. They act as coenzymes in critical metabolic pathways, including energy production (B1), amino acid metabolism (B6), and lipid synthesis (biotin) [24]. Their inclusion in defined media for metazoans is a standard practice to prevent deficiencies and support optimal growth. The complete chemical composition and preparation protocol of SRM are listed in Table 1. The prepared SRM was diluted as needed prior to use, and the optimal dilution factors were determined experimentally.

(2) Lettuce juice:

Fresh lettuce was thoroughly washed and processed in a juicer, with 1 L of tap water/distilled water added per batch. The extracted juice was distributed into glass bottles and sterilized by autoclaving (FLS-1000, TOMY, Tokyo, Japan).

(3) Flour medium:

Wheat flour (0.1 g/L) was added to sterile water to prepare a flour medium.

All media were prepared according to aseptic principles. Sterilization was performed using a 0.22 μm membrane filter within a biological safety cabinet. A serial dilution series (10×, 50×, 100×, 500×, 1000×, and 10,000×) with five biological replicates per concentration was used, to determine the optimal SRM concentration for culturing *A. vaga*. Five juvenile *A. vaga* specimens were obtained from the SRM pre-cultures following the method described by Xiang et al. [25] and randomly allocated into the wells of a 24-well plate. One milliliter of diluted SRM was added to each well. The plates were incubated at 20 °C in a constant-temperature incubator (RXZ-5000, Shuangxu, Shanghai, China). Daily rotifer counts were conducted using a stereomicroscope (SAPO, LEICA, Wetzlar, Germany), and the medium was refreshed every three days. Lettuce juice and flour medium served as controls.

### 2.3. Evaluation Indices

Population growth curves and specific growth rates (SGR) were used to evaluate the population growth characteristics of *A. vaga* grown on different media.

The specific growth rate was calculated as follows:

SGR = (ln N_t_ − ln N_0_)/t (1)

where SGR (d^−1^) is the specific growth rate of the bdelloid rotifer, N_t_ (ind./mL) is the abundance after time t, N_0_ (ind./mL) is the initial abundance, and t (days) is the culture time interval.

### 2.4. Isolation and Identification of A. vaga-Associated Bacteria

To ensure that the bacterial strains represented consistent host associations, rather than transient or idiosyncratic occurrences, a replicate sampling strategy was implemented across multiple independent cultures. Five independent culture lines (>30-day-old wells, each containing > 100 rotifers) were selected as biological replicates. More than 30 rotifers were randomly selected from each replicate for sequential processing.

For epizoic bacteria, each selected *A. vaga* individual was rinsed five times separately with sterile water and transferred to a 1.5 mL centrifuge tube containing 1 mL sterile water. The bacteria attached to the body surface were eluted by ultrasonic treatment (ultrasonic frequency: 40 KHz; ultrasonic power: 600 W) for 2–5 min. The integrity of the body after treatment was monitored using a dissecting microscope (SAPO, LEICA, Germany) to prevent the release of bacteria inside the rotifers due to excessive ultrasonic treatment. The eluent was processed for microbial isolation.

For endozoic bacteria, selected *A. vaga* individuals after ultrasonic treatment (ultrasonic frequency: 40 KHz; ultrasonic power: 600 W) were transferred to a 0.001% NaClO solution for 5–10 s to kill the bacteria attached to the body surface [26]. Each rotifer individual was subsequently rinsed five times with sterile water. To confirm the effectiveness of the surface sterilization, the final rinse was checked using the plate spreading method; no bacterial growth was observed (Figure S1). This treatment caused *A. vaga* to contract. All these steps were performed under continuous observation using a dissecting microscope to prevent the rotifer body from being dissolved or damaged by oversoaking in the NaClO solution (Figure S1). The treated *A. vaga* specimens were transferred to 1.5 mL centrifuge tubes containing 1 mL sterile water after surface cleaning with sterile water. Bacterial release was achieved via mechanical disruption using a sterile grinding rod.

The samples containing associated bacteria were serially diluted (10^−1^, 10^−2^, 10^−3^, 10^−4^, 10^−5^, and 10^−6^), and 100 μL bacterial solution was taken from each gradient and coated on the pre-prepared nutrient agar medium, with three replicates, then cultured in a constant-temperature incubator at 20 °C. After seven days, distinct bacterial colonies were selected based on variations in their macroscopic morphology, including size, shape, color, margin, elevation, and surface texture. Each morphologically distinct colony was purified separately by streaking onto two fresh nutrient agar plates using an inoculation loop. The purified strains were stored in 20% glycerol at −80 °C for long-term preservation.

DNA extraction from single bacterial colonies utilized a Chelex resin protocol, a widely adopted technique for efficient microbial DNA isolation [27], and their 16S rRNA genes were amplified using primers 27F (5′-AGAGTTTGATCCTGGCTCAG-3′) and 1492R (5′-AAGTCGTAACAAGGTARCCGTA-3′). PCR amplification products were detected using 1% agarose gel electrophoresis, and qualified samples were sent to TsingkeBiotechnology Co., Ltd. (Guangzhou, China) for sequencing. Sequencing results were aligned with known sequences from the EzBiocloud database https://www.ezbiocloud.net/ (accessed on 19 February 2024) to identify the species. A phylogenetic tree was constructed using MEGA 7.0 based on the neighbor-joining method [28].

### 2.5. Antibiotics Treatment

**Figure 1:**
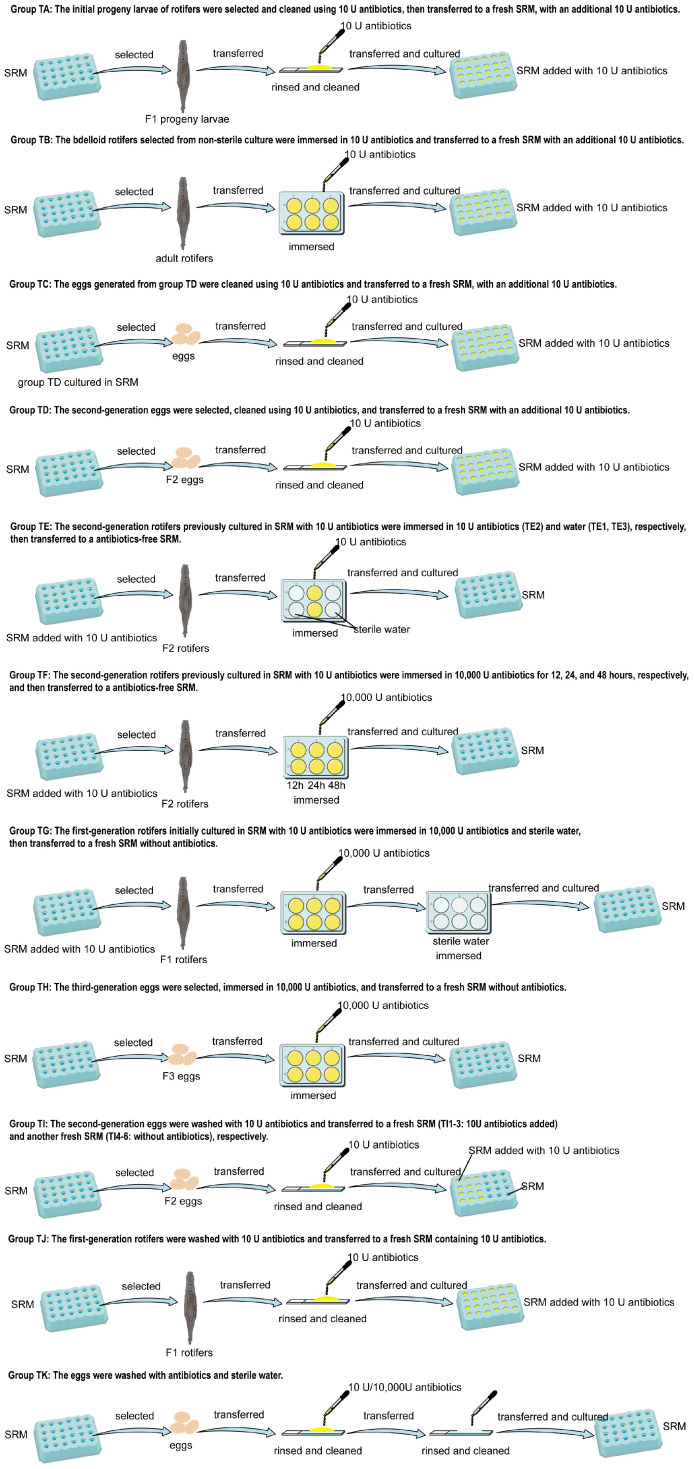
Antibiotic Treatment Protocol.

To understand the impact of antibiotic addition on the population growth of bdelloid rotifers and their associated bacteria, penicillin-streptomycin-amphotericin B was used to treat bdelloids based on preliminary experiment results. Eleven antibiotic treatments (TA–TK, Figure 1) were tested to eliminate rotifer-associated bacteria, and their effects on the 20-day population growth of *A. vaga* in SRM were evaluated, starting from five individuals per group. These antibiotic treatments were divided into two groups: continuous culture with antibiotic addition and culture after antibiotic immersion (Figure 1). After 20 days of cultivation for each treatment group, the population and SGR of the bdelloid rotifers were measured. Plate spreading and molecular methods were used to detect the presence of associated bacteria in the medium and bdelloid rotifers to test the inhibitory effect of antibiotics on bacteria associated with bdelloid rotifers. For plate spreading detection, 100 μL of the medium after 20 days of culture was spread onto pre-prepared nutrient agar medium and incubated at 20 °C for 3–7 days for detection; Additionally, 50 bdelloid rotifers from each treatment were collected, transferred to 1.5 mL sterile EP tubes, and ground with a sterile pestle. Plate spread detection was conducted as described in Section 2.4. Three replicates and control groups were used for all the treatments. For molecular detection, 50 individuals from the high-abundance treatment groups were collected and ground. DNA was extracted using the HOTSHOT method [29]. This protocol induced immediate dissolution of *A. vaga* specimens upon immersion in lysis buffer, concurrently releasing both endozoic (internal) and epizoic (surface-associated) bacteria into the solution. However, treatments with low rotifer abundance yielded insufficient DNA for downstream analysis. Target gene fragments were amplified by PCR using the primers listed in Table 2. Electrophoretic separation of the products was performed on 1.5% agarose gels, followed by documentation using a GelDoc XR+ imaging system (Bio-Rad, Hercules, CA, USA).

### 2.6. Statistical Analysis

The population growth dynamics of *A. vaga* across different SRM concentrations and control groups (natural food-supplemented media: lettuce juice/wheat flour) were characterized using line plots. SGRs were compared using box plots, with intergroup differences assessed using the Kruskal–Wallis and Mann–Whitney tests. Post-20-day antibiotic treatments—Group F (culture after antibiotic immersion) and Group C/I (continuous culture with antibiotic addition)—were evaluated against controls using box plots for population density and SGR, with Kruskal–Wallis tests determining significance. All multiple comparisons were performed using Dunn’s post hoc test. Normality assumptions were verified prior to the analyses, and all visualizations and statistical procedures were performed using GraphPad Prism 6.01.

## 3. Results

### 3.1. Establishment of Synthetic Media and Corresponding Culture Methods for Bdelloid Rotifer A. vaga

**Figure 2:**
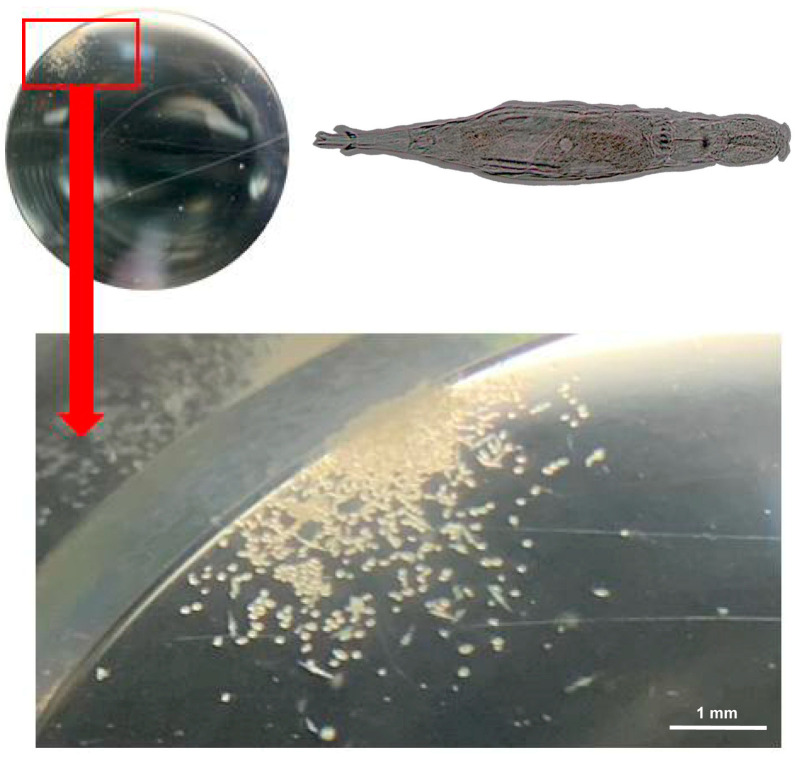
The population size of *A. vaga* cultured in SRM for 20 days (detected using a stereomicroscope). The yellow ovals are the eggs, and the yellow spindles are the crawling rotifers. The inset in the upper-right corner shows a detailed morphological view of an *A. vaga* individual.

In this study, an SRM was developed based on the BG-11 medium. Compared to the original BG-11 medium, glucose, sucrose, fructose, vitamins B1, B6, and D-biotin were added, while EDTA-Na_2_ was removed. The effects of SRM on *A. vaga* cultures are shown in Figure 2.

**Figure 3:**
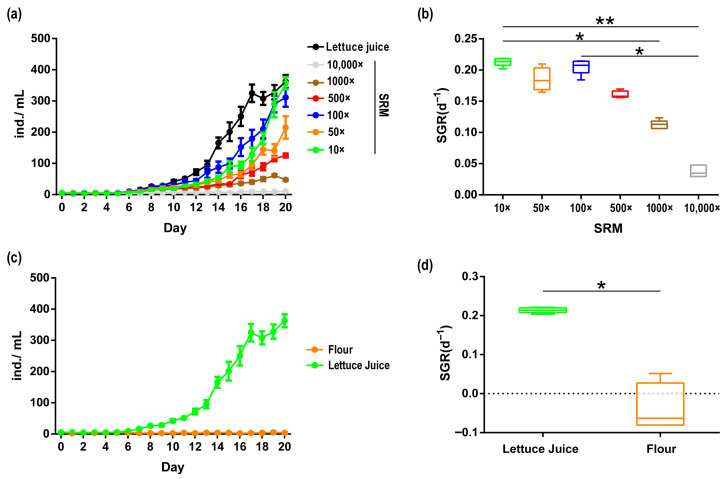
(**a**) Population growth curve of *A. vaga* cultured in SRM with different concentrations and lettuce juice. (**b**) Specific growth rate (SGR) of *A. vaga* cultured in SRM with different concentrations. (**c**) Population growth curve of *A. vaga* cultured in lettuce juice and flour medium. (**d**) Specific growth rate (SGR) of *A. vaga* cultured in lettuce juice and flour medium. Error bars denote min–max range; * *p* < 0.05, ** *p* < 0.001.

Different concentrations of SRM resulted in varying cultivation effects on *A. vaga* (Figure 3). Except for the 10,000× diluted SRM, *A. vaga* cultured in other concentrations of medium entered the reproductive stage on the 6th day. On the 12th day of cultivation, the *A. vaga* population cultured in the 100× diluted SRM had the largest population size (44.4 ± 5.537 ind./mL) and the highest SGR (0.1798 ± 0.009). The population growth curve showed that 10× and 100× diluted SRM had the best effect. On the 20th day of cultivation, the abundance of *A. vaga* cultured in the 10× diluted SRM (357 ± 19.95 ind./mL) exceeded that of those cultured in the 100× diluted SRM (311.4 ± 29.79 ind./mL); however, there was no significant difference in their SGRs (Kruskal–Wallis test, *p* > 0.05). In lettuce juice (control group), the population of *A. vaga* entered a rapid growth phase on the 12th day, slightly declined on the 18th day, and ultimately showed results similar to those of the 10× diluted SRM on the 20th day. Its SGR (0.2138 ± 0.003) showed no significant difference compared to those of the 10× diluted SRM (0.2131 ± 0.003) or the 100× diluted SRM (0.2055 ± 0.006) (Kruskal–Wallis test, *p* > 0.05) (Figure 3). The SGR in the 10× diluted SRM was significantly higher than in the 1000× and 10,000× dilutions (Kruskal–Wallis test, *p* < 0.05). Similarly, the 100× diluted SRM exhibited a higher SGR than the 10,000× dilution (Kruskal–Wallis test, *p* < 0.05). No statistically significant differences were observed among the other dilution groups (Kruskal–Wallis test, *p* > 0.05). In contrast, the traditional wheat flour-supplemented system (control) demonstrated the poorest performance, with measurable population decline observed during cultivation (SGR: −0.0388 ± 0.03119). Critically, both the 10× diluted SRM (357 ± 19.95 ind./mL) and lettuce juice control groups (362.6 ± 20.92 ind./mL) showed comparable growth performance after 20 days of cultivation, surpassing those of all other tested media (Figure 3). In summary, we established a chemically defined SRM that sustained robust population growth in *A. vaga* without natural food supplementation, achieving performance parity with traditional systems while eliminating compositional variability.

### 3.2. Isolation and Identification of Bdelloid Rotifer-Associated Bacteria

**Figure 4:**
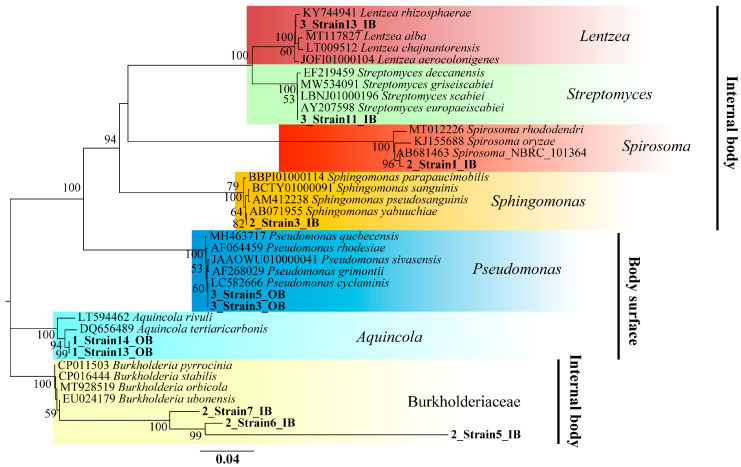
Phylogenetic analysis of 16S rRNA gene sequences from epizoic and endozoic bacterial strains of the bdelloid rotifer *A. vaga* based on the neighbor-joining method.

A total of 20 bacterial strains were successfully isolated from *A. vaga* cultured in SRM, with 11 endozoic strains originating from internal tissues and nine epizoic strains colonizing the body surface (Figure 4) (Table 3). The phylogenetic taxonomy at the class level revealed diverse community compositions, including Betaproteobacteria (35%), Gammaproteobacteria (25%), Actinomycetia (20%), Alphaproteobacteria (15%), and Cytophagia (5%). Based on the 16S rRNA gene sequencing analysis, the endozoic strains were phylogenetically assigned to four genera (*Lentzea*, *Streptomyces*, *Spirosoma*, and *Sphingomonas*) and the family Burkholderiaceae, whereas the epizoic strains predominantly comprised two genera (*Pseudomonas* and *Aquincola*) (Figure 4). The sequence alignment analysis using the EzBiocloud database revealed that 17 isolated strains (85%) exhibited ≥98.7% 16S rRNA gene sequence similarity to validated type strains, enabling species-level identification. The remaining three strains (15%) showed limited sequence similarities (75.37–93.43%) and were provisionally classified at the family level (Burkholderiaceae) (Table 3). All 20 pure-cultured isolates were cryopreserved at −80 °C for long-term storage. We characterized the identity and localization of culturable bacterial strains associated with *A. vaga* in this study, thereby establishing a valuable resource for future investigations of rotifer–microbe interactions.

### 3.3. Effect of Antibiotic Treatment on Bdelloid Rotifers and Their Associated Bacteria

**Figure 5:**
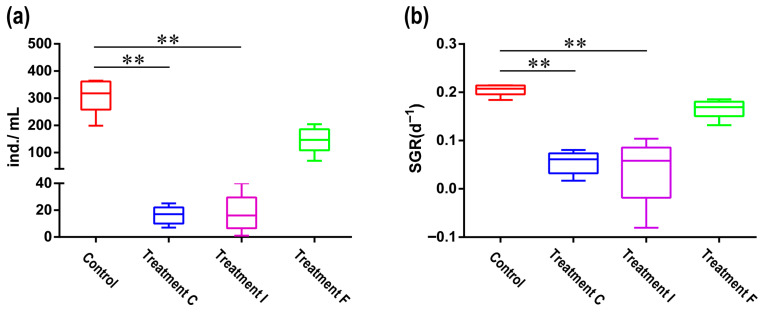
(**a**) Population size of *A. vaga* under different antibiotic protocols. Treatment F: Acute high-concentration exposure (10,000 U/mL, 12–24 h); Treatment C/I: chronic low-concentration treatments (10 U/mL, 20 days). (**b**) The effects of antibiotic treatment on the specific growth rate (SGR) of *A. vaga*. Error bars denote min-max range; ** *p* < 0.001.

Antibiotic exposure markedly suppressed population dynamics in most treatment groups of the bdelloid rotifer *A. vaga*. Notably, a complete population collapse occurred in the TE group (no surviving individuals), whereas the TA, TC, and TI groups retained only a small population (<10 individuals). Prolonged low-concentration treatments (TC/TI: 10 U/mL over 20 days) significantly reduced both population size (TC: 16.2 ± 3.007; TI: 17.6 ± 6.377) and SGR (TC: 0.0546 ± 0.011;TI: 0.0384 ± 0.031) compared to those in the controls (abundance: 311.4 ± 29.79; SGR: 0.2055 ± 0.006) (Kruskal–Wallis test, *p* < 0.01) (Figure 5). In contrast, acute high-concentration exposure (TF: 10,000 U/mL for 12–24 h) showed no statistically discernible impact on demographic parameters (abundance: 147.2 ± 21.93; SGR: 0.1662 ± 0.009) relative to untreated cohorts (Kruskal–Wallis test, *p* > 0.05).

**Figure 6:**
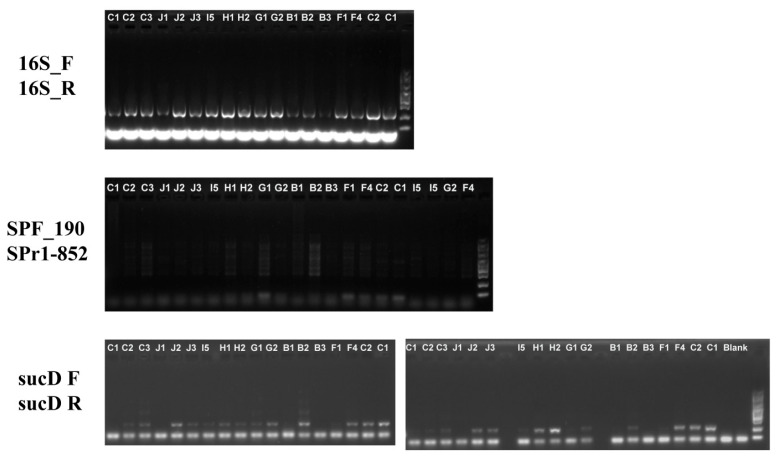
Detection of bacteria associated with *A. vaga* using PCR amplification.

Microbiological cultivation assays revealed persistent bacterial colonization in most of the treatment groups (Figure S2a). Critically, TA, TE, and TK groups exhibited complete bacterial eradication in the medium, concomitant with severe *A. vaga* population decline (TA: ≤2 moribund individuals; TE/TK: no motile rotifers detected). Nevertheless, direct plating of rotifer-associated microbiota from the TC, TF, TH, and TI groups confirmed residual bacterial viability (Figure S2b), indicating incomplete symbiont clearance despite the antibiotic regimens. In support of this, 16S rRNA PCR amplification confirmed the non-axenic status of antibiotic-treated *A. vaga*. Notably, *Sphingomonas* band intensity was significantly attenuated. In contrast, *Pseudomonas* persisted despite antibiotic exposure, suggesting that penicillin–streptomycin–amphotericin B cocktails preferentially inhibited specific taxa, but failed to eradicate persistent colonizers (Figure 6). In summary, the current antibiotic-based protocol is ineffective for generating axenic rotifers. The treatment suppressed rotifer population growth and failed to completely eradicate the associated bacteria.

## 4. Discussion

### 4.1. Advantages and Applications of the SRM

Recent studies in genomics [12,33], stress physiology [34,35,36,37], and biogeography of cryptic species [38,39] have established bdelloid rotifers as important model organisms. The application of technologies such as comparative genomics and transcriptomics in rotifer research relies on high-density cultures, prompting the development of SRM, a nutritionally autonomous, chemically defined medium (i.e., no additional food needs to be added) that enables population expansion while avoiding the “black box” limitations inherent in undefined natural media supplemented with natural foods.

In the present study, SRM supplemented with defined carbohydrates (glucose, fructose, and sucrose) effectively proliferated the *A. vaga* population, producing a large number of viable offspring. The cultivation efficiency and number of offspring produced were comparable to those obtained using lettuce juice medium or synthetic medium supplemented with natural food. Even when the medium was diluted tenfold and cultured for 20 days, the *A. vaga* population remained in the logarithmic growth phase, demonstrating the potential to surpass the effects of the lettuce juice medium (Figure 3). In contrast, Ricci [14] used *E. coli*, yeasts *D. hansenii* and *S. capricornutum* as food to cultivate *A. vaga*, with population densities not exceeding 100 individuals by the 20th day and SGRs of 0.114 ± 0.021, 0.141 ± 0.005, and 0.104 ± 0.007, respectively. He et al. [15] developed and evaluated a flour-based medium for cultivating the bdelloid rotifer *Habrotrocha* sp., isolated from activated sludge in a wastewater treatment plant. By the 12th day of cultivation, the population density of *H*. sp. exceeded 100 individuals, with an SGR of 0.55. Additionally, feeding with microalgae (*Chlorella vulgaris*) and bacteria (mixed bacteria) resulted in SGRs of 0.39 and 0.5, respectively, which were different from those of *H. elusa vegeta* [14]. However, in the present study, using flour to cultivate *A. vaga* proved ineffective, resulting in partial rotifer mortality. This discrepancy may be due to differences in rotifer species, feeding methods (*H.* sp.: filtering-feeding; *A. vaga*: scraping-feeding), and environmental origins (*H.* sp.: activated sludge; *A. vaga*: moss on soil). The superior culturing efficiency of SRM is likely attributable to its direct alignment with *A. vaga*’s specific nutritional requirements for carbon sources and vitamins, avoiding both nutrient deficiency and toxic accumulation. In contrast, natural media formulations may exhibit nutritional imbalances or redundancy. Furthermore, natural media carry the inherent risk of introducing biogenic inhibitors, including algal-derived phycotoxins, phenolic compounds, and opportunistic pathogens (e.g., parasitic fungi), which can disrupt feeding behavior and metabolic homeostasis [40,41,42]. Moreover, the nutritional composition of natural food sources may change during cultivation, making them unsuitable for nutritional evaluation in animal studies. The axenic nature of SRM eliminates these confounding variables, thereby enhancing culture stability and experimental reproducibility. The feeding efficiency of bdelloid rotifers is critically dependent on particle size selectivity [15]. The fully water-soluble composition of SRM ensures optimal nutrient bioavailability, whereas particulate heterogeneity in natural media or food compromises feeding performance. Moreover, *A. vaga*’s osmolality sensitivity necessitates precise ionic regulation, a key advantage of SRM formula that maintains ionic homeostasis within physiologically tolerable ranges, in contrast to the inherent batch-to-batch variability of natural media. The inclusion of osmoprotectants, such as sucrose, has been demonstrated to stabilize biomacromolecular structures and membrane integrity during desiccation-rehydration cycles [43]. Notably, several longevity-enhancing compounds with proven antioxidative and fitness-increasing effects, including α-tocopherol (vitamin E) [35], trehalose [43], and kynurenic acid [44], have not been incorporated into SRM formulations. Strategic supplementation of these biomolecules could further enhance rotifer population dynamics, highlighting SRM’s modular design and its advantage for iterative optimization and hypothesis-driven customization of culture parameters.

Nevertheless, culture methods based on natural or synthetic media supplemented with natural food are still the predominant strategy for the rapid cultivation of bdelloid rotifers [45]. This is primarily due to the substantial developmental costs and formulation-optimization challenges associated with chemically defined synthetic media without natural food. Therefore, some studies have added natural foods to the culture protocols. McCarthy et al. [46] tested the suitability of a soil elutriate and balanced salt solution (BSS) for culturing Antarctic soil rotifers. Although the BSS medium is primarily composed of common laboratory ingredients, it requires the addition of *Chlorella* sp. as food during cultivation, which undoubtedly complicates the media composition. Similar media designs are common in many rotifer cultivation systems such as the US EPA medium (food: microalgae) and Pourriot-Gilbert medium (food: paramecia) [11,47,48,49]. Additionally, sterile procedures and techniques can effectively prevent the population decline caused by fungal contamination [50]. It has also been reported that aseptic manipulation can stabilize the mean life span of bdelloid rotifers [47]. Unlike known media, SRM completely avoids the issue of undefined food components and is the first synthetic medium without natural food for bdelloid rotifers. This provides favorable conditions for controlled indoor studies on bdelloid rotifers, thereby avoiding the selective pressure imposed by exogenous food on host-associated microorganisms. Consequently, it facilitates a clearer understanding of the relationships among bdelloid rotifers, microbial symbiosis, and energy metabolism, offering advantages and application prospects that existing media do not possess. Notably, this novel SRM also supports the cultivation of planktonic Monogononta rotifers (e.g., *Brachionus plicatilis* Müller, 1786) inhabiting open-water environments, as verified in our preliminary experiments (unpublished data). Its applicability to broader zooplankton taxa indicates its potential for use in aquaculture and environmental monitoring.

### 4.2. The Limitations of SRM and Future Directions

The SRM group exhibited a slower population growth phase during the first 17 days of cultivation than the lettuce juice control group. This primarily reflects the necessary adaptation process as rotifers transition from a complex natural diet to a chemically defined dissolved nutrient source, a phenomenon consistently observed in all SRM culture experiments. Although the SRM group showed slow initial growth, it eventually reached a population density comparable to that of the lettuce juice control, demonstrating that SRM can provide sufficient and balanced nutrition to support long-term population maintenance once the rotifers have adapted.

Furthermore, when examining the effects of different SRM concentrations on rotifer population growth, a non-monotonic dose–response was observed among the 10×, 50×, and 100× dilution groups. This pattern arose because of the dynamic changes in the amount of nutrients available per individual rotifer and to the population as a whole as the *A. vaga* population grew. Over time, the initially optimal 100× dilution may have become nutrient-limited, whereas the originally supra-optimal 10× dilution may have supplied sufficient nutrients to sustain population growth. The intermediate 50× dilution supported rapid population initiation, but it could not maintain growth throughout the cultivation period, leading the rotifers to cycle between satiety and starvation. This “feast-famine cycling” likely has more severe negative impacts on long-term population health (e.g., reproduction and survival) than either continuous saturation or continuous limitation. These results underscore the importance of identifying an optimal concentration window for chemically defined medium, such as SRM, and highlight a key difference in cultivation dynamics between SRM and traditional natural food sources: the former requires more precise concentration optimization.

Notably, the generalizability of our findings may be influenced by the specific SRM formulations. Although it supports robust population growth, the defined composition may lack certain, as-yet-unidentified, micronutrients or growth factors present in natural diets, such as lettuce juice. This could provide an additional explanation for the initial growth lag observed in SRM cultures compared to that in the control. Consequently, the optimal formulation presented here may require further tailoring for different rotifer species, living environments, and specific research applications. For example, in ecotoxicology, interactions between medium components and stressors must be avoided, whereas in genetic studies, undefined nutritional gaps can skew gene expression profiles. Therefore, future work should test the SRM across a wider range of rotifer species and refine its composition by systematic comparisons with natural media under different experimental scenarios.

### 4.3. Potential Functions of A. vaga-Associated Bacteria

Using the SRM, we isolated epizoic and endozoic bacterial strains associated with the bdelloid rotifer *A. vaga* for the first time. This preliminary study documents the diversity of rotifer-associated bacteria and provides a bacterial resource for future mechanistic studies of rotifer–microbe interactions in this system. Notably, *Pseudomonas* was the most dominant taxon among the isolated strains, adhering to the surface of *A. vaga*, which is consistent with the results of 16S amplicon sequencing of the rotifer-associated microbiome [20]. These findings confirmed the universality of *Pseudomonas* in rotifers. Numerous studies have confirmed the benefits of *Pseudomonas* in bdelloid rotifer survival, including the provision of vitamin B12 [47] and the secretion of substances that antagonize fungal infections [50,51]. This antifungal function is likely related to its location on the body surface, enabling the timely prevention of fungal invasion. The best-matching species from the EzBiocloud database, *Pseudomonas rhodesiae*, was also found in the Asian tiger mosquito *Ae. albopictus* and possesses antiviral capabilities in vitro [52]. The other two isolated strains, *Sphingomonas* and Burkholderiaceae, were also detected using 16S amplicon sequencing of rotifer-associated microbiomes [20]. These bacteria can metabolize organic compounds, providing carbon, nitrogen, and other nutrients to the host, and potentially participate in *A. vaga*’s nutrient absorption and energy metabolism [53,54]. Bacteria belonging to the Burkholderiaceae family are common symbionts of various microscopic animals. They confer nutrient and pesticide resistance to stinkbugs; protect *Lagria* beetle eggs from pathogens; and provide essential amino acids, B vitamins, and waste-recycling services to bean bugs [55,56]. Furthermore, *Burkholderia* can induce closure of the bean bug midgut to prevent pathogen invasion [57]. The *Burkholderia*-amoeba system is an excellent model for studying host-bacterium symbiosis. Amoebae can incorporate beneficial *Burkholderia* via phagocytosis, whereas bacteria migrate toward the amoeba supernatant via chemotaxis to establish a stable symbiotic relationship [58,59]. Additionally, *Burkholderia* can induce phenotypic changes in amoebae, enhancing their capacity to carry food bacteria [60]. *Sphingomonas* is frequently associated with various microscopic invertebrates such as benthic nematodes and oligochaetes [61], and can be transmitted transgenerationally as symbionts in the insect *Steingelia gorodetskia* [62]. *Sphingomonas* has been reported to be associated with bioflocculants produced by rotifers, nematodes, and ciliates, which are beneficial to host health [63]. Successful isolation of these bacteria confirmed their dominance in *A. vaga*.

*Aquincola* strains colonize the body surface of *A. vaga* and can degrade various organic compounds, such as synthetic dialkyl ethers [64], to form bioflocs [65]. However, interactions between *Aquincola* and their microscopic hosts have not yet been reported. Similarly, although *Lentzea aerocolonigenes* and *Streptomyces griseiscabiei* have been isolated from the interior of *A. vaga*, research on their relationships with hosts has primarily focused on their interactions with plant hosts. Notably, *Streptomyces* are considered plant pathogens [66]. Based on our bacterial identification results, the endozoic and epizoic bacterial communities associated with *A. vaga* cultured in SRM exhibited distinct differences. This clear taxonomic separation suggests niche-specific colonization and potential functional specialization between internal and surface-associated bacteria. Notably, the persistent association of these bacterial taxa with *A. vaga* even under chemically defined synthetic culture conditions highlighted their potential importance as key microbial partners that may play beneficial roles in host physiology. However, the contributions of these bacteria to rotifer fitness require further investigation. It should be noted that the objective of using isolation and cultivation methods in this study was to obtain bdelloid rotifer-associated bacterial strain resources.

Prior studies have confirmed the intimate interactions between bdelloid rotifers and microorganisms, including the provision of essential nutrients and enhancement of the environmental adaptability of the rotifer host. These isolated bacterial strains will serve as a valuable resource for future rotifer–microbe co-culture experiments, aiming to identify core beneficial microbes that promote rotifer growth, and thereby elucidate the underlying interaction and coevolution mechanisms. This represents a preliminary investigation into the diversity of bacteria associated with bdelloid rotifers. A more comprehensive characterization of this microbial community and its functional roles will require the application of complementary methods: (1) utilizing high-throughput sequencing techniques such as 16S rRNA gene amplicon sequencing and metagenomic sequencing to analyze the diversity and functional potential of the rotifer-associated bacterial community; (2) conducting co-culture experiments with the isolated bacterial strains and the host *A. vaga* to evaluate their fitness effects, including impacts on host population growth, lifespan, fecundity, and physiological parameters, while integrating multi-omics approaches (transcriptomics, metabolomics, and genomics) to elucidate the underlying interaction mechanisms; (3) using fluorescent protein-labeled bacterial strains for in vivo microscopic localization to determine the precise micro-interfaces of these interactions. In summary, our study provides initial insights into the potential symbiotic bacteria of *A. vaga* and identifies their specific localization in or within the rotifer body.

### 4.4. Development Attempts of Axenic A. vaga and Its Significance in Rotifer–Microbe Interaction Research

The development of an axenic rotifer host model, which is similar to axenic amoebae, represents a fundamental solution to resolve interference from diverse host-associated bacteria in rotifer–microbe interaction experiments [67]. Such an axenic host system would effectively eliminate biological interference in research on rotifer–microbe interactions, HGT, and environmental adaptation. By providing a clean background, it enables focused investigation of specific target relationships while excluding both direct and indirect confounding effects from the host’s intrinsic bacterial community. This axenic system enables in-depth mechanistic investigations, the precise determination of response pathways, and the critical transition from correlative inference to causal demonstration. This is particularly critical for testing long-standing hypotheses on how microbial associations influence key evolutionary processes in bdelloids, such as the integration of horizontally transferred genes [6,7] and the genetic basis of their extraordinary adaptation to stressors like desiccation and radiation [5,18]. The addition of antibiotics is one of the primary methods used to reduce the bacterial load carried by hosts and to prepare axenic model hosts [60,68]. Previous studies have found that the addition of antibiotics can decrease host fitness [69] or alter host development and phenotype [70]. Identifying suitable treatment concentrations and methods to balance the growth and development of bdelloid rotifers with effective bacterial eradication is crucial. Although the use of penicillin–streptomycin–amphotericin B in this study did not completely eliminate bacteria associated with bdelloid rotifers, it appeared to inhibit *Sphingomonas*. Future studies could reintroduce *Sphingomonas* into the *A. vaga* culture system to test the interactions between these bacteria and the bdelloid rotifer hosts. However, the associated bacteria that were not removed may be necessary for the life activities of the bdelloid rotifers. However, failure to completely eradicate bacteria, coupled with the potential negative impact of antibiotics on rotifer health, presents a complex interpretive challenge. It is plausible that residual bacteria provide unrecognized nutritional benefits or that antibiotic stress impairs rotifer physiology, thereby confounding the true metabolic pathways and interaction effects between rotifer and target bacteria. Notably, in comparison to the control group, bacteria emerged after 20 d of bdelloid rotifer cultivation in SRM, suggesting that bdelloid rotifers might serve as vectors for their successful dispersal and colonization in new environments, which is similar to the farming effect of amoebae carrying symbiotic bacteria [60]. These findings suggest two possible reasons: (1) antibiotic inefficacy: current dosage/duration protocols insufficiently target rotifer-associated bacterial consortia; (2) microbial resilience: protected niches (e.g., intracellular reservoirs) enable bacterial persistence, with subsequent vertical transmission during medium renewal cycles.

Current research on rotifer–microbe interactions has predominantly focused on the relationship between probiotic supplementation and the growth performance of rotifers used as live feed in aquaculture, highlighting that predatory interactions are the most direct pathway for microbial entry into rotifers [71]. In bdelloid rotifers, HGT at the genomic level is a critical mechanism that enhances environmental adaptability [6,7]. However, there remains a significant knowledge gap in understanding the processes and mechanisms underlying survival, mutualistic symbiosis, and commensalism between rotifers and their colonizing microbes, bridging the realms of predation and gene transfer. Addressing these questions necessitates integrated approaches that combine laboratory cultivation experiments with multi-omics technologies. The development of a new synthetic medium without natural food for bdelloid rotifers is the basis for establishing a solid model system for research on bdelloid rotifer–microbe interactions. Using this system, we successfully acquired a stable *A. vaga* strain and pure cultures of associated bacteria. This resource, combined with the future achievement of axenic cultures, will allow researchers to reconstitute defined microbial communities and directly test their role in shaping rotifer phenotypes and evolutionary trajectories. For example, it would be possible to investigate whether specific bacteria facilitate the expression of horizontally acquired genes, contribute to stress resilience through metabolic exchange, or drive co-evolutionary adaptations. Currently, such axenic host models remain scarce and have only been successfully established in a limited number of microscopic organisms, such as amoebae and microalgae [58,67,68]. Nevertheless, these models have proven crucial for understanding the evolution and adaptation of species. Therefore, the development of a reliable axenic rotifer model would represent a significant advancement, providing a unique opportunity to investigate fundamental questions in host–microbe coevolution and environmental adaptation within a previously inaccessible metazoan system. However, axenic bdelloid rotifers have yet to be cultivated. Most bdelloid rotifers are sensitive to environmental changes and lack a protective cuticle. Chemical treatments, such as sodium hypochlorite and 75% alcohol solutions, or physical methods, such as ultrasonication, are unsuitable and can cause dormancy or even death. Notably, ultraviolet irradiation has been reported to eliminate 99% of surface-associated bacteria of rotifers without adverse effects on their viability, and warrants further exploration [72]. Nevertheless, the methods for producing axenic rotifers require further study. Although such attempts are challenging, they are crucial for studying rotifer–microbe interactions and their mechanisms.

## 5. Conclusions

An SRM with chemically defined constituents, excluding natural food inputs, was developed for effective *A. vaga* cultivation and demonstrated yields comparable to those of natural food-supplemented protocols. In addition, we isolated 20 endozoic and epizoic *A. vaga* strains grown in SRM. Additionally, the addition of low-concentration antibiotics over 20 days reduced the population sizes or SGRs, and could not fully eliminate the associated bacteria of *A. vaga*. This study provides valuable tools and resources for further understanding the molecular mechanisms of rotifer–microbe interactions and evolution.

## Figures and Tables

**Table 1:** Components and preparation methods of SRM.

Component Category	Compound Name	Stock Solution Concentration (g/L)	Working Concentration (g/L)	The Dosage Required to Prepare 1 L Working Solution (mL)	Preparation Method
Basic components	NaNO_3_ (Analytical reagent, Aladdin^®^, Shanghai, China)	150	1.5	10	Stock solutions were prepared separately, stored at 4 °C protected from light, and appropriate amounts were aliquoted to prepare the working solution prior to use.
	K_2_HPO_4_ (Analytical reagent, Aladdin^®^)	40	0.04	1	
	MgSO_4_·7H_2_O (Analytical reagent, Aladdin^®^)	75	0.075	1	
	CaCl_2_·2H_2_O (Analytical reagent, Aladdin^®^)	36	0.036	1	
	Na_2_CO_3_ (Analytical reagent, Aladdin^®^)	20	0.02	1	
	citric acid (Analytical reagent, Aladdin^®^)	6	0.006	1	
	ferric ammonium citrate (Analytical reagent, Aladdin^®^)	6	0.006	1	
trace elements	H_3_BO_3_ (Analytical reagent, Aladdin^®^)	2.86	0.00286	1	Individual components were weighed, dissolved in 1 L of pure water with stirring to prepare the stock solution, from which 1 mL was aliquoted into another 1 L of pure water to prepare the working solution.
	MnCl_2_·4H_2_O (Analytical reagent, Aladdin^®^)	1.81	0.00181	1	
	ZnSO_4_·7H_2_O (Analytical reagent, Aladdin^®^)	0.222	0.000222	1	
	Na_2_MoO_4_·2H_2_O (Analytical reagent, Aladdin^®^)	0.39	0.00039	1	
	CuSO_4_·5H_2_O (Analytical reagent, Aladdin^®^)	0.079	0.000079	1	
	Co(NO_3_)_2_·6H_2_O (Analytical reagent, Aladdin^®^)	0.0494	0.0000494	1	
Carbon Sources	glucose (99%, Macklin^®^, Shanghai, China)	/	1	/	The working solution was prepared by directly adding appropriate amounts of these components, omitting the pre-preparation of stock solutions.
	sucrose (99%, Macklin^®^)	/	1	/	
	fructose (99%, Macklin^®^)	/	1	/	
Vitamins	vitamin B1 (98%, Macklin^®^)	/	0.005	/	
	vitamin B6 (99%, Macklin^®^)	/	0.005	/	
	D-biotin (98%, Macklin^®^)	/	0.005	/	

**Table 2:** Primers for the detection of bacteria associated with bdelloid rotifers.

Primer	Target Bacteria	Reference
16S_F (5′-CGGCCCAGACTCCTACGGGAGGCAGCAG-3′) 16S_R (5′-GCGTGGACTACCAGGGTATCTAATCC-3′)	all bacteria	Govendir et al. [30]
sucD F (5′-CGTCCTGATCAATAAAGACACC-3′) sucD R (5′-GATGCAGACGATCAGCTTG-3′)	*Pseudomonas*	Zhang et al. [31]
SPF_190 (5′-MRGWCCAAAGATTTATCG-3′) SPr1-852 (5′-CMAADCACCAWGTGMCCKGA-3′)	*Sphingomonas*	Leung et al. [32]

**Table 3:** Identification of epizoic and endozoic bacterial strains of *A. vaga* based on EzBiocloud database.

Location	Family/Genus	Top-Hit Taxon	Number of Strain	Similarity (%)
Endozoic bacteria	*Lentzea*	*Lentzea aerocolonigenes*	2	99.06–99.21
	*Streptomyces*	*Streptomyces griseiscabiei*	2	99.71–99.78
	Burkholderiaceae	*Burkholderia orbicola/Burkholderia ubonensis/Burkholderia singularis*	3	75.37–93.43
	*Sphingomonas*	*Sphingomonas sanguinis*	3	99.78
	*Spirosoma*	*AB681463_s*	1	99.34
Epizoic bacteria	*Pseudomonas*	*Pseudomonas rhodesiae*	5	99.85–99.86
	*Aquincola*	*MIMtkpLc11*	4	99.86–99.78

## Data Availability

The 16S rRNA gene sequences of isolated bdelloid rotifer-associated bacterial strains have been deposited in GenBank under accession numbers PV915616 to PV915624. The other data are available from the corresponding author on reasonable request.

## Supplementary Materials

The following supporting information can be downloaded at: https://www.mdpi.com/article/10.3390/biology14111507/s1, Figure S1: (a) Examination of rotifer body integrity after treatment with 0.001% NaClO solution. (b) Validation of the 0.001% NaClO treatment protocol for eliminating epizoic bacteria; Figure S2: (a) Detection of bacteria in the SRM using the spread plate method. (b) Detection of bacteria associated with *A. vaga* using the spread plate method.
