# Supplementary material for: Cultivation of Bdelloid Rotifer Adineta vaga with Synthetic Medium and Characterization of Associated Bacteria
Source: Biology (Basel). 2025 Oct 28;14(11):1507. doi: 10.3390/biology14111507 (PMC12650600; doi:10.3390/biology14111507)
Supplement: Supplementary file 1 [file biology-14-01507-s001.zip › biology-3872919-supplementary.pdf]

Supplemental Information for:

Cultivation of Bdelloid Rotifer *Adineta vaga* with Synthetic Medium and Characterization of Associated Bacteria

Wenbo Wang, Zhili He, Qing Wang and Yufeng Yang

Table of Contents

**Supplementary Figure S1**	Page 2
**Supplementary Figure S2**	Page 3

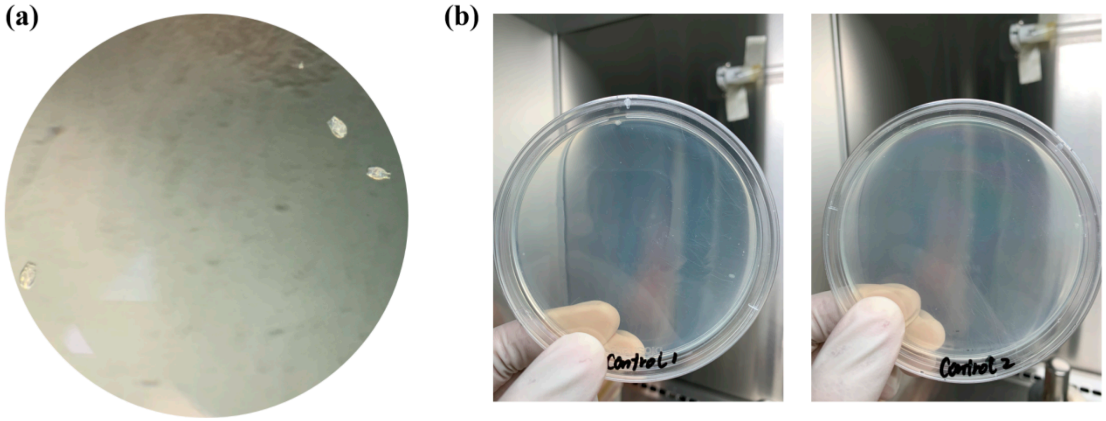

Figure S1 (a) Examination of rotifer body integrity after treatment with 0.001% NaClO solution. (b) Validation of the 0.001% NaClO treatment protocol for eliminating epizoic bacteria.

(a)

➤ Treatment A

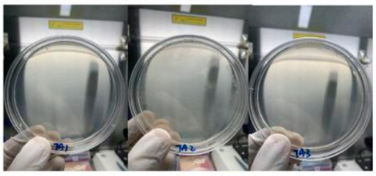

➤ Treatment B

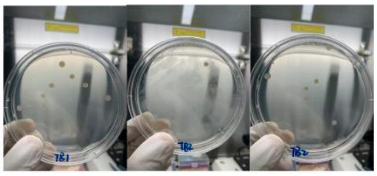

➤ Treatment C

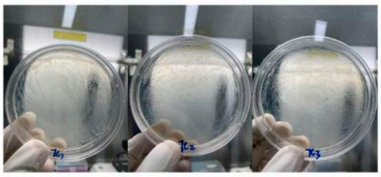

➤ Treatment D

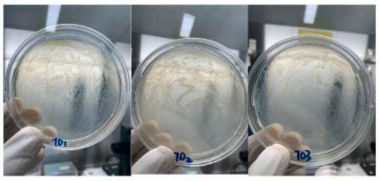

➤ Treatment E

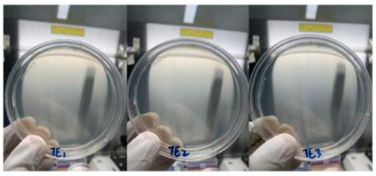

➤ Treatment F

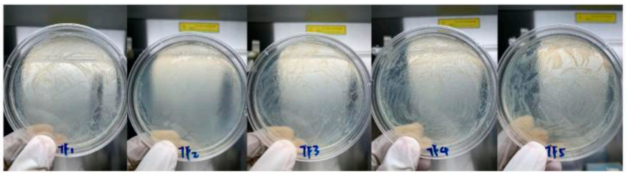

➤ Treatment G

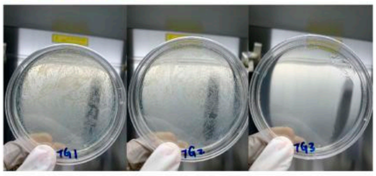

➤ Treatment H

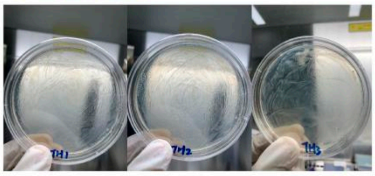

➤ Treatment I

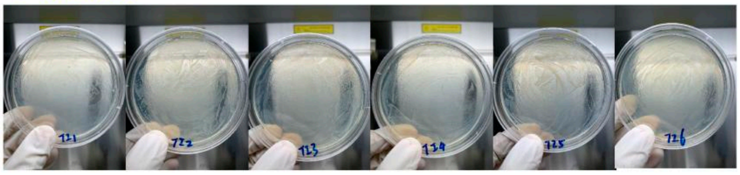

➤ Treatment J

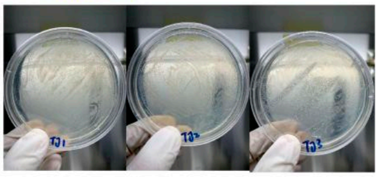

➤ Treatment K

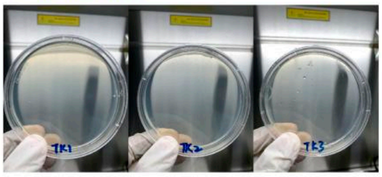

(b)

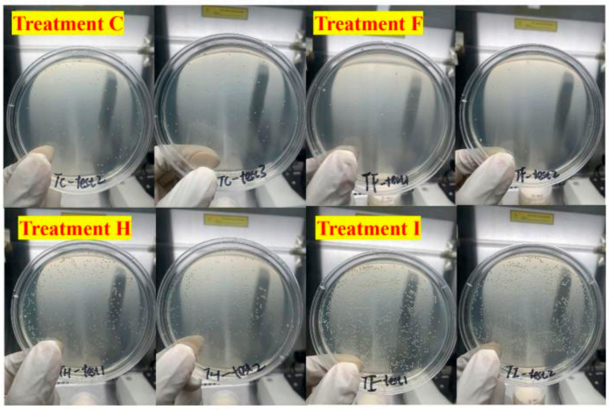

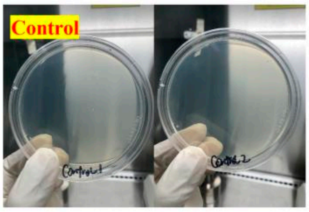

Figure S2 (a) Detection of bacteria in the SRM using the spread plate method. (b) Detection of bacteria associated with *A. vaga* using the spread plate method.
